# Shallow entangled circuits for quantum time series prediction on IBM devices

**DOI:** 10.1038/s41598-025-28512-6

**Published:** 2025-12-12

**Authors:** Mostafizur Rahaman Laskar, Richa Goel

**Affiliations:** 1 IBM Quantum, IBM Research Lab, Bangalore, India; 2 IBM Quantum, IBM Research Lab, Gurgoan, India

**Keywords:** Mathematics and computing, Physics

## Abstract

Forecasting temporal dynamics underpins many areas of science and engineering, from large-scale atmospheric prediction to nanoscale quantum control. Classical approaches, including autoregressive models and deep neural networks, have advanced sequential learning often at the expense of known model order, or large dataset and parameters, resulting in computational cost. Here, we investigate whether quantum entanglement can serve as a resource for temporal pattern learning using shallow and structured quantum circuits. We have proposed a Quantum Time Series (QTS) framework that encodes normalised sequential data into single-qubit rotations and captures temporal correlations through forward and cross-entanglement layers. Among several encoding schemes, phase encoding-based sparse entanglement provides hardware efficiency by scaling to larger qubit systems with linear circuit depth and two-qubit complexity of $\mathcal {O}(n)$ for qubit size *n*. This offers a reduction in parameters and depth compared with deep variational quantum circuits such as Heisenberg-inspired circuits, and random-parametric unitary architectures. Experiments on synthetic and geophysical datasets show that shallow QTS circuits reproduce complex temporal pattern from limited data by leveraging structured quantum entanglement. Executions on IBM’s Heron and Eagle-class processors demonstrate robustness and scalability up to 100 qubits. These results suggest that structured entanglement may offer a short-term memory effect for time-series analysis, providing a scalable route for near-term quantum applications.

## Introduction

Time series forecasting is a crucial and challenging task for predicting future trends and inferring predicted outcomes based on historical data, which is essential for informed decision-making across various scientific and technological domains at diverse scales. In the geophysical domain, numerical weather and climate systems rely on the accurate extrapolation of complex spatiotemporal patterns, necessitating models that strike a balance between predictive accuracy, interpretability, and computational efficiency^1,2^. At microscopic scales, simulating electronic, vibrational, and spin dynamics remains essential for materials discovery, reaction control, and the characterisation of quantum devices^3^. Finance and healthcare datasets, among many others, are important and require precise forecasting. However, the intrinsic heterogeneity, nonlinearity, and high dimensionality of real-world dynamical systems continue to challenge the expressivity, generalizability, and scalability of current models. Classical statistical frameworks, such as autoregressive (AR), moving-average (MA), and ARIMA models^4^, provide efficient and interpretable representations of linear processes but may lose fidelity when extended to chaotic dynamics, high-dimensional systems, or when the model order is unknown. In contrary, deep learning architectures, including long short-term memory (LSTM) networks^5^, gated recurrent units (GRUs)^6^, temporal convolutional networks (TCNs)^7,8^, and attention-based Transformer models^9,10^, have considerably improved the modeling of long-range and multiscale dependencies with large number of parameters. Further, these models typically require large training datasets, extensive parameterisation, and significant computational resources, which limit their applicability in data-sparse or low-latency forecasting contexts. As an alternative approach, we investigate whether quantum phenomena, such as entanglement, can leverage temporal dynamics with fewer parameters.

Quantum computational models, which leverage superposition and entanglement, have been studied for machine learning problems under constrained data and parameter budgets^11^. These parameterised quantum circuits (PQCs) employ various data encoding ansatzes, such as Haar-random and general unitary ansatz^12,13^. However, their practical realisation is often hindered by the overhead of hybrid quantum–classical optimisation (the classical computational limit for parameter optimisation) and by the emergence of barren plateaus, sometimes in the loss function.

In this work, we investigate various non-parametric (or fixed-parameter) and parametric variants (where the parameters are optimised from a hybrid learning model, such as ridge regression) of unitary time evolution circuits for forecasting tasks. As a core objective of the work, entanglement is exploited as a computational resource for mimicking temporal patterns in the QTS task. In the fixed-parametric variant, normalised scalar inputs are mapped onto single-qubit rotation gates (with fixed-rotational angles), while temporal coupling is exploited through deterministic forward and cross-entangling layers. This architecture systematically increases representational power from rotational encoding, by $R_y(\theta )$ gates, to forward-neighbour entanglement, $R_y(\theta )$+Fwd, and full forward plus cross entanglement, $R_y(\theta )$+Fwd+Cross, while maintaining linear circuit depth and two-qubit complexity of $\mathcal {O}(n)$ for qubit size *n*. The proposed QTS framework enables direct execution on near-term quantum devices without iterative variational training. We benchmark multiple QTS variants (both fixed-parameter and trainable-parameter models) against classical baselines, including AR, ARIMA, TCN, Transformer, and compact multilayer perceptron (MLP) models, using synthetic noisy AR datasets, chaotic dynamical data, and meteorological time series. Through noisy simulations and hardware experiments on IBM Quantum superconducting processors, we demonstrate that structured, shallow entangled unitary circuits can reproduce complex temporal behaviour with limited reduced data and fewer parameters.

### Contributions

- We propose a Quantum Time Series (QTS) framework that provides an interpretable, quantum-native analogue of autoregressive sequence modelling. The framework maps normalised temporal windows onto single-qubit rotations and encodes inter-temporal dependencies through sparse, structured entanglement. The proposed model exhibits short-term memory-like behaviour for the forecasting task, as evaluated on a noisy synthetic dataset and a real weather forecasting dataset.
- We have explored several strategies for efficient quantum encoding ansatz to exploit temporal correlation. Among various encoding ansatz, the $R_y(\theta )$+Fwd and $R_y(\theta )$+Fwd+Cross circuits are shallow and hardware-efficient. Furthermore, these sparse and patterned entanglements progressively enhance circuit expressivity while maintaining low error rates, making them suitable for time-series data on noisy near-term hardware. We have demonstrated a shallow circuit (with approximately hundreds of CNOT gates) in the 100-qubit regime on IBM quantum hardware, which does not incorporate any error-mitigation techniques in the current study.
- We have presented extensive empirical results for benchmarking of the proposed QTS models in the presence of classical baselines such as AR, ARIMA, TCN, Transformer, and MLP on synthetic and real meteorological datasets. Further, we demonstrate the practical executability of the proposed QTS models on IBM Heron-class and Eagle-class processors. An analysis of two-qubit gate counts and circuit depth for various qubit sizes is also presented to check the scalability of the presented approach.

## Quantum time series model

We propose a shallow quantum encoding ansatz for univariate time series forecasting. Let $\{x_t\}_{t \in \mathbb {Z}}$ be a normalized univariate time series with $x_t \in [0,1]$. To forecast $x_{t+1}$, we construct an input vector $\textbf{x}_t = [x_{t-n+1}, \dots , x_{t}]^{\top } \in \mathbb {R}^n$ from *n* consecutive past observations. Each scalar $x_{t-i+1}$ for $i=1,\dots ,n$ is encoded into an *n*-qubit quantum circuit via single-qubit *Y*-rotations with angle $\theta _{i} = 2\pi x_{t-i+1}$, producing the initial product state given by

1 $$\begin{aligned} |\Psi _t\rangle = \bigotimes _{i=1}^{n} R_y(2\pi x_{t-i+1})|0\rangle _i. \end{aligned}$$

To augment the temporal correlations across encoded features, we apply a fixed entangling unitary $U_{\text {ent}}$ consisting of two classes of CNOT gates. The forward coupling $U_{\text {Fwd}}$ comprises CNOT$(i \rightarrow i+1)$ for $i = 1, \dots , n-1$, connecting adjacent qubits. The cross coupling $U_{\text {Cross}}$ applies CNOT$(i \rightarrow i+l)$ for selected indices *i* with skip length *l* satisfying $2 \le l \le n-i$, establishing longer-range connections; for instance, $l=2$ yields CNOT$(i \rightarrow i+2)$. The composite unitary $U_{\text {ent}} = U_{\text {Fwd}}U_{\text {Cross}}$ transforms the state as follows

2 $$\begin{aligned} |\Psi _{\text {ent}}\rangle = U_{\text {ent}} |\Psi _t\rangle . \end{aligned}$$

This construction maintains circuit depth $\mathcal {O}(n)$, linear in the number of qubits. Forecasting proceeds through projective measurement of $|\Psi _{\text {ent}}\rangle$ in the computational basis $\{|k\rangle \}_{k=0}^{2^n-1}$. Measurement outcome *k* occurs with probability given by $P(k) = |\langle k | \Psi _{\text {ent}} \rangle |^2$. The predicted value is computed as the normalized expectation given by $\hat{x}_{t+1} = \sum _{k=0}^{2^n - 1} \frac{k}{2^n - 1} \cdot P(k)$.

For enhanced expressivity, we extend the model with trainable parameters. The encoding unitary becomes

$$\begin{aligned} U_{\text {rot}}(\varvec{\theta }, \textbf{x}_t) = \bigotimes _{i=1}^{n} R_y(\theta _i\,x_{t-i+1}), \end{aligned}$$

where $\varvec{\theta } = \{\theta _i\}_{i=1}^n$ are learnable rotation amplitudes. Following encoding, we apply an entangling layer parameterized by $\varvec{\phi } = \{\phi _i\}_{i=1}^{n-1}$ as $U_{\text {ent}}(\varvec{\phi }) = \prod _{i=1}^{n-1} \exp \!\left( -i\, \phi _{i}\, Z_i\, Z_{i+1}\right)$. The parameterized quantum state is then given by

3 $$\begin{aligned} |\Psi _t \rangle = U_{\text {ent}}(\varvec{\phi }) \cdot U_{\text {rot}}(\varvec{\theta }, \textbf{x}_t)\; |0 \rangle ^{\otimes n}, \end{aligned}$$

where the complete parameter set comprises the rotational encoding angles $\varvec{\theta }$ and entangling angles $\varvec{\phi }$. This parametric variant follows the framework of variational quantum algorithms^14^, and needs to depend on hybrid quantum and classical workload. Hence, fixed parametric variants are scalable on quantum hardware.

### Nonlinear temporal correlations through entanglement

The structured entanglement operation encodes multi-lag temporal correlations through quantum amplitude interference. We formalize this observation as follows.

#### Proposition 1

Let $\textbf{x}_t = [x_{t-n+1}, \dots , x_{t}]^{\top } \in [0,1]^n$ and define the initial product state $|\Psi _t\rangle = \bigotimes _{i=1}^{n} R_y(2\pi x_{t-i+1}) |0\rangle _i$. Let $U_{\textrm{ent}}$ be the structured entangling unitary. The measurement probability on the final state $|\Psi _{\textrm{ent}}\rangle = U_{\textrm{ent}} |\Psi _t\rangle$, given by $P(k) = |\langle k | \Psi _{\textrm{ent}} \rangle |^2$ is a nonlinear function of $\textbf{x}_t$ containing cross-terms of the form $\cos (\pi x_{t-i+1}) \sin (\pi x_{t-j+1})$ for $i \ne j$, thereby encoding distributed temporal dependencies beyond linear superposition.

#### Proof

The initial product state can be expanded in the computational basis as

$$\begin{aligned} |\Psi _t\rangle&= \bigotimes _{i=1}^{n} \left[ \cos (\pi x_{t-i+1})|0\rangle _i + \sin (\pi x_{t-i+1})|1\rangle _i \right] ~= \sum _{k=0}^{2^n-1} \alpha _k^{(0)} |k\rangle , \end{aligned}$$

where *k* labels the computational basis state $|k\rangle = |b_1 b_2 \cdots b_n\rangle$ with $b_i \in \{0,1\}$, and $\alpha _k^{(0)} = \langle k | \Psi _t \rangle$ denotes the corresponding probability amplitude in the initial state. For this product state, the amplitude factorizes as $\alpha _k^{(0)} = \prod _{i=1}^{n} a_i^{(b_i)}(x_{t-i+1})$, where each factor takes the form $a_i^{(0)}(x_{t-i+1}) = \cos (\pi x_{t-i+1})$ if the *i*-th qubit is in state $|0\rangle$, or $a_i^{(1)}(x_{t-i+1}) = \sin (\pi x_{t-i+1})$ if it is in state $|1\rangle$. Critically, each factor depends solely on a single time index, making $\alpha _k^{(0)}$ a simple product of independent trigonometric functions. Consequently, the measurement probability before entanglement, $P^{(0)}(k) = |\alpha _k^{(0)}|^2$, contains no cross-terms between different time indices.

The CNOT operation $\textrm{CNOT}(i \rightarrow j) = |0\rangle _i \langle 0| \otimes I_j + |1\rangle _i \langle 1| \otimes X_j$ couples qubits *i* and *j*, breaking the factorization structure. Applying the structured unitary $U_{\text {ent}}$ transforms the state to

$$\begin{aligned} |\Psi _{\textrm{ent}}\rangle = U_{\text {ent}}|\Psi _t\rangle = \sum _{k=0}^{2^n-1} \alpha _k |k\rangle , \end{aligned}$$

where the new amplitude $\alpha _k = \langle k | \Psi _{\textrm{ent}} \rangle$ contains inference terms in its representation due to the permutation action of CNOT gates on computational basis states. Considering forward coupling CNOT$(i \rightarrow i+1)$ acting on the state for a given target basis state $|k\rangle$, the amplitude $\alpha _k$ accumulates contributions from multiple initial states, each with factorized structure. This produces terms such as $\cos (\pi x_{t-i+1})\sin (\pi x_{t-i})$, coupling adjacent time indices. Similarly, cross coupling CNOT$(i \rightarrow i+l)$ with $l \ge 2$ generates products coupling $x_{t-i+1}$ with $x_{t-i-l+1}$, extending correlations across longer temporal distances. After applying the complete entangling unitary $U_{\text {ent}}$, the amplitude $\alpha _k$ takes the general form

$$\begin{aligned} \alpha _k = \sum _{j \in \mathcal {J}_k} c_j \prod _{\ell \in S_j} g_\ell (x_{t-\ell +1}), \end{aligned}$$

where $\mathcal {J}_k$ indexes the contributing computational paths, $c_j \in \mathbb {C}$ are coefficients determined by the CNOT connectivity pattern, $S_j \subseteq \{1,\dots ,n\}$ with $|S_j| \ge 2$ denotes the set of coupled time indices, and $g_\ell \in \{\cos (\pi x_{t-\ell +1}), \sin (\pi x_{t-\ell +1})\}$ selects the appropriate trigonometric function.

Computing the measurement probability yields

4 $$\begin{aligned} P(k) = |\alpha _k|^2 = \alpha _k \alpha _k^* = \sum _{j,j' \in \mathcal {J}_k} c_j c_{j'}^* \prod _{\ell \in S_j} g_\ell (x_{t-\ell +1}) \prod _{m \in S_{j'}} g_m^*(x_{t-m+1}). \end{aligned}$$

This expression contains quantum interference terms where $j \ne j'$, producing nonlinear cross-products such as

$$\begin{aligned} \cos (\pi x_{t-i+1})\sin (\pi x_{t-j+1}) \cos (\pi x_{t-k+1}), \end{aligned}$$

for distinct temporal indices *i*, *j*, *k*. These nonlinear cross-terms cannot be expressed as sums of univariate functions, confirming that *P*(*k*) encodes multi-variate temporal correlations. The structured pattern of $U_{\text {ent}}$ systematically couples time indices $(t-i+1, t-i)$ through forward gates and $(t-i+1, t-i-l+1)$ through cross gates with skip length $l \ge 2$, embedding distributed temporal information across the quantum state. $\square$

### Comparison with alternative encoding ansatz

Alternative quantum encoding strategies exhibit different trade-offs. Amplitude encoding maps an *N*-dimensional vector $\textbf{x}$ directly onto state amplitudes as $|\psi \rangle = \sum _{i=0}^{N-1} x_i |i\rangle$ using $n=\lceil \log _2 N \rceil$ qubits, offering exponential compression. However, generic state preparation requires circuit depth $\mathcal {O}(2^n)$^15^. Haar-random unitaries from $\mathcal {U}(2^n)$ provide maximal expressivity but demand depth $\mathcal {O}(n^2)$, and sometimes making them susceptible to barren plateaus^12,13^. Heisenberg-inspired circuits based on XXZ spin-chain Hamiltonians achieve linear depth $\mathcal {O}(N)$ but require iterative variational optimization^16^. Random entanglement patterns via unstructured gates offer high entanglement without geometric structure, potentially leading to training instabilities^13^.

The present architecture combines structured $R_y$ encoding with sparse, geometrically-motivated entanglement. The fixed-parameter variant eliminates variational training overhead while maintaining $\mathcal {O}(n)$ depth, facilitating stable implementation on current quantum hardware. However, one can do trade-off between complexity and expressivity.

### Classical readout layer

For predictions, we employ Ridge regression on quantum measurement statistics. At each time step, the quantum circuit prepares $|\Psi _t\rangle$, which is sampled *R* times to estimate expectation values of Pauli observables. We define the feature vector $\varvec{\xi }_t \in \mathbb {R}^d$ as

$$\begin{aligned} \xi _{t,j} = \langle \Psi _t | \mathcal {O}_j | \Psi _t \rangle , \quad j = 1, \dots , d, \end{aligned}$$

where each observable $\mathcal {O}_j$ can be a Pauli operator (e.g., $Z_j$) or a product thereof (e.g., $Z_i Z_j$). The estimator primitive can be used here for the estimation of the parameters. From *T* training samples, we construct the design matrix $\textbf{X} \in \mathbb {R}^{T \times d}$ with rows $\varvec{\xi }_t^\top$ and target vector $\textbf{y} \in \mathbb {R}^T$ containing values $x_{t+1}$. The prediction is given by

$$\begin{aligned} \hat{x}_{t+1} = \textbf{w}^\top \varvec{\xi }_t + b, \end{aligned}$$

where weights $\textbf{w} \in \mathbb {R}^d$ and bias $b \in \mathbb {R}$ are obtained by minimizing

5 $$\begin{aligned} \min _{\textbf{w}, b} \left\{ \Vert \textbf{X} \textbf{w} + b \textbf{1} - \textbf{y}\Vert ^2 + \lambda \Vert \textbf{w}\Vert ^2 \right\} , \end{aligned}$$

with regularization parameter $\lambda > 0$. This readout strategy shares conceptual similarities with quantum reservoir computing^17–19^, but employs shallow circuits and direct projective measurements, enabling scalability to large qubit numbers on noisy intermediate-scale quantum devices with limited classical post-processing overhead.

The fixed-parametric ansatz variant contains no variational quantum parameters, eliminating gradient-based circuit optimization. The parametric extension requires training the classical readout weights via Ridge regression.

## Results

We evaluate the fixed-parametric QTS model through systematic experiments on synthetic and real-world data. Performance is assessed via rolling one-step-ahead forecasts with mean squared error as the primary metric. All quantum circuits are simulated using exact statevector methods to isolate algorithmic performance from hardware noise.

### Datasets and evaluation protocol

Three time series capture different temporal dynamics. The synthetic autoregressive (AR) follows $x_t = 0.8\,x_{t-1} + 0.2\,u_t +\varepsilon _t$, where $u_t$ is uniformly distributed in [0, 1] and $\varepsilon _t \sim \mathcal {N}(0,\sigma ^2)$ denotes Gaussian noise. After generation, we normalise the series to [0, 1] via min-max scaling. This series provides a baseline for linear temporal encoding.

We additionally evaluate on the *Mackey-Glass system*, a time-delay differential equation modelling chaotic dynamics, similar to Ref.^17^, given by

$$\begin{aligned} \frac{dx(t)}{dt} = \beta \,\frac{x(t-\tau )}{1 + x(t-\tau )^{n}} - \gamma \,x(t), \end{aligned}$$

where the parameters $\beta = 0.2$, $\gamma = 0.1$, $n = 10$, and $\tau = 17$. We integrate this system using the fourth-order Runge-Kutta method with a time step of $dt=1.0$, discard the initial transient regime, and normalise the results to the interval [0, 1]. For verification of the proposed models on the real datasets, we have taken an atmospheric dataset from ERA-type data containing geopotential height at the 500 hPa pressure level. We spatially average the global field over latitude and longitude to obtain a one-dimensional time series, scaled by $10^{-4}$ for numerical stability, and normalise to [0, 1]. We employ a rolling forecast protocol where at each time step *s*, we construct a training segment of length $\text {TRAIN\_LEN} + L$ (where *L* is the lag window size matching the qubit count), extract overlapping windows $\textbf{x}^{(i)}=[x_i,x_{i+1},\dots ,x_{i+L-1}]$ with targets $y^{(i)}=x_{i+L}$, fit the model on this segment, and predict the next value $x_{s+\text {TRAIN\_LEN}+L}$. The window then slides forward by one time step. This procedure generates one-step-ahead forecasts over a specified horizon.

For SNR robustness experiments, we vary noise standard deviation $\sigma$ and define $\textrm{SNR}_{\textrm{dB}} = 10\log _{10}(1/\sigma ^2)$. Experiments are repeated over multiple random seeds to ensure statistical reliability. Specific configurations for *simulated experiments* include: 8 qubits (lag window $L=8$) for most noise robustness and comparison studies, training window length of 8 time steps, forecast horizons of 10 to 100 steps depending on the experiment, SNR values from $-10$ to 6 dB tested at intervals, and 3 to 10 Monte Carlo trials per configuration. For the Mackey-Glass comparison, we evaluate different window sizes ($L \in \{4, 8, 12\}$) and forecast horizons ($h \in \{1, 10, 50\}$) using 5 qubits, with 500 total time steps allocated 67% for training and 33% for testing. For *hardware experiments*, we scale to 10 and 100 qubits to assess practical scalability on IBM quantum processors, with 100 training points and forecast horizons of 15 to 20 steps.

Quantum circuits in simulation are executed using Qiskit statevector methods, providing exact probability distributions without shot noise to isolate algorithmic performance. The scalar readout $\hat{x}_{t+1} = \sum _{k=0}^{2^n-1} \frac{k}{2^n-1} P(k)$ is computed from measurement probabilities $P(k) = |\langle k|\Psi _{\text {ent}}\rangle |^2$, and an affine calibration $(a, b_0)$ is fitted per training window via ridge regression (regularization $\alpha =10^{-6}$) to map quantum readouts to the target scale. For hardware experiments, circuits are transpiled to native gate sets, executed with shot-based sampling (typically 1024 to 4096 shots), and noise characteristics are inherited from the specific backend. Classical baselines include autoregressive models (AR(1), AR(2), AR(3), ARIMA(2, 1, 0)), support vector regression with RBF kernel (SVR), kernel ridge regression with RBF kernel, multilayer perceptrons with 32 hidden units (MLP), small temporal convolutional networks (TCN), and a compact feedforward network (Tiny MLP). All predictions are clipped to [0, 1] for fair comparison. An additional quantum baseline, employing a fixed ZZ feature map ($R_y$ rotations followed by $R_{ZZ}$ gates with fixed angles), is combined with ridge regression on Pauli expectation values to contrast structured entanglement with generic feature extraction.

### Performance on noisy AR(1) dataset (synthetic)

**Fig. 1:**
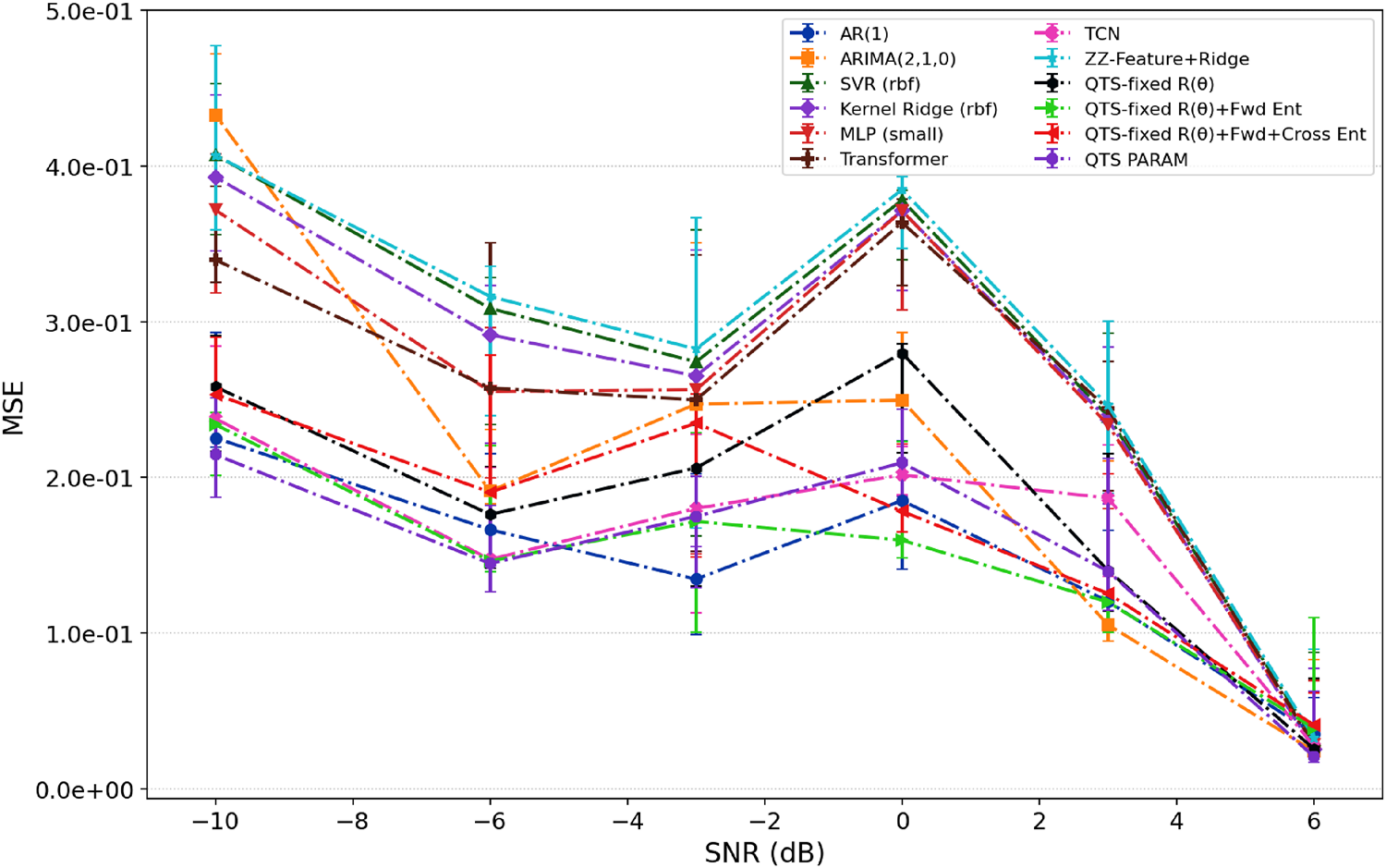
Performance comparison on the noisy AR(1) time series. The plot shows the mean squared error (MSE) achieved by each model (classical and quantum) as a function of the Signal-to-Noise Ratio (SNR), measured in decibels (dB). Error bars indicate the inter-quartile range (IQR) across multiple Monte Carlo trials, reflecting the statistical stability of the models under varying noise conditions.

Figure 1 presents a signal-to-noise ratio (SNR) sweep for the noisy AR(1) process, evaluated across classical and quantum forecasting methods. Across all models, median MSE decreases monotonically with increasing SNR, declining from approximately $(2\times 10^{-1}$ to $4.7\times 10^{-1})$ at $-10$ dB to approximately $(2.0\times 10^{-2}$ to $5.0\times 10^{-2})$ at $+6$ dB. This order-of-magnitude improvement is consistent with the expected behavior under additive Gaussian noise, where prediction accuracy scales with signal quality. At high SNR ($+6$ dB), classical autoregressive models $\textrm{AR}(1)$ and $\textrm{ARIMA}(2,1,0)$ achieve the lowest median errors, typically (2.5 to $3.5)\times 10^{-2}$, reflecting their strong suitability for linear temporal dynamics. Support vector regression (SVR) and kernel ridge regression follow closely in the (3.0 to $4.5)\times 10^{-2}$ range. At mid-range SNR ($-6$ to 0 dB), these methods exhibit median errors in the (1.3 to $3.1)\times 10^{-1}$ band, with modest inter-quartile spreads indicating consistent performance across multiple trials. The neural network baselines such as MLP, Transformer, and TCN follow comparable SNR–MSE profiles, clustering near classical curves at high SNR but showing increased sensitivity around $-2$ to 0 dB, where median errors reach approximately (2.0 to $3.7)\times 10^{-1}$. The inter-quartile ranges narrow at high SNR and widen moderately near 0 dB, reflecting heightened sensitivity to signal quality in intermediate noise regimes.

The quantum predictors maintain competitive accuracy throughout the SNR range. The $\mathrm {ZZ\text {-}Feature{+}Ridge}$ baseline tracks the best-performing classical methods at high SNR, achieving median errors approximately $3.0\times 10^{-2}$ at $+6$ dB. The fixed-parameter QTS variants with entanglement structures $\mathrm {QTS\text {-}fixed}\,R(\theta )$ (rotations only), $R(\theta ){+}\textrm{Fwd}$ (forward entanglement), and $R(\theta ){+}\mathrm {Fwd{+}Cross}$ (forward and cross entanglement ($2^{nd}$ order) achieve approximately (3.0 to $4.5)\times 10^{-2}$ at $+6$ dB and (1.7 to $2.8)\times 10^{-1}$ near 0 dB. Among these, the forward+cross entanglement pattern demonstrates the highest stability, as evidenced by tighter error bars across the SNR range. The compact parametric variant (QTS PARAM) achieves median errors comparable to the fixed circuits and exhibits slightly narrower uncertainty bands at higher SNR values, suggesting modest benefit from trainable parameters in low-noise conditions. Figure 1 demonstrates that both classical and quantum predictors follow consistent monotonic SNR–MSE scaling. Notably, the $R(\theta ){+}\textrm{Fwd}$ architecture maintain competitive accuracy while preserving shallow circuit depth for AR(1) prediction task.

### Forecasting chaotic Mackey-Glass dynamics (synthetic)

**Fig. 2:**
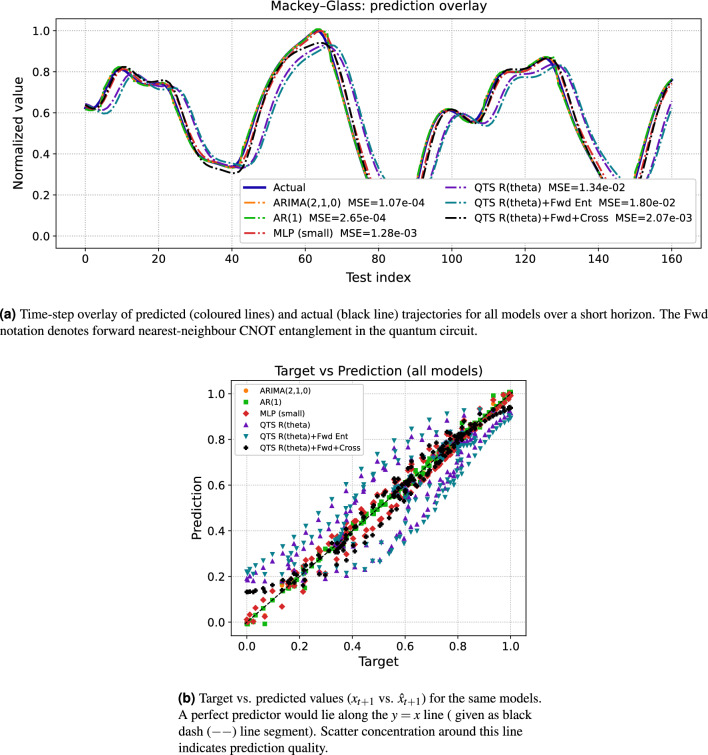
Prediction performance analysis on the Mackey-Glass chaotic series. The experiment utilized a lag length $L=4$ (encoded on 5 qubits, $n_q=5$) with a one-step forecast horizon. Classical baselines ($\textrm{ARIMA}$, $\textrm{AR}(1)$, and $\textrm{MLP}$) are quantitatively compared against three quantum circuit architectures: $R(\theta )$ (rotational encoding only), $R(\theta )+\text {Fwd}$ (nearest-neighbour entanglement), and $R(\theta )+\text {Fwd+Cross}$ (extended entanglement).

Figure 2 presents one-step forecasting performance on the Mackey-Glass chaotic time series, a benchmark system for evaluating nonlinear prediction methods. All models were trained on two-thirds of the trajectory and evaluated on the remaining one-third using lag length $L=4$ (encoded on 5 qubits) with a one-step forecast horizon. The trajectory overlay in Fig.  2a demonstrates that the quantum temporal sampling architectures successfully reproduce the smooth, bounded oscillations characteristic of the Mackey-Glass attractor. Classical baselines achieve excellent performance: $\textrm{ARIMA}(2,1,0)$ attains $\textrm{MSE}=1.07\times 10^{-4}$, $\textrm{AR}(1)$ reaches $2.65\times 10^{-4}$, and the small MLP achieves $1.28\times 10^{-3}$. Among quantum variants, the cross-entangled model ($\textrm{QTS}\,R(\theta )+\mathrm {Fwd{+}Cross}$) delivers the strongest performance with correlation $r=0.983$ and $\textrm{MSE}=2.07\times 10^{-3}$, representing a twenty-fold improvement over the rotation-only circuit ($1.34\times 10^{-2}$) and a nine-fold improvement over forward entanglement alone ($1.80\times 10^{-2}$). This hierarchy–where cross entanglement substantially outperforms simpler architectures–contrasts with the more modest differences observed for the linear AR(1) process (Fig.  1), indicating that skip-layer couplings provide enhanced expressivity specifically for capturing nonlinear temporal structures.

The scatter plot in Fig.  2b confirms these quantitative findings through visual inspection of prediction quality. Data points from classical models (ARIMA, AR(1)) cluster tightly along the diagonal $y=x$ line, reflecting their high fidelity. The quantum models exhibit progressively tighter concentration with increasing entanglement: $R(\theta )$ alone shows significant scatter, $R(\theta )+\text {Fwd}$ narrows substantially, and $R(\theta )+\text {Fwd{+}Cross}$ approaches classical baseline clustering, considering up to $2^{nd}$ order cross entanglement. This progression demonstrates that shallow quantum circuits with structured entanglement patterns can effectively learn deterministic chaotic dynamics despite restricted fixed-parameterization. The results establish that for nonlinear temporal systems, the specific connectivity of entangling gates plays a critical role in model expressivity, with cross entanglement offering a favorable trade-off between circuit depth (remaining $\mathcal {O}(n)$) and representational capacity for complex temporal correlations.

### Forecasting performance on the real atmospheric dataset

**Fig. 3:**
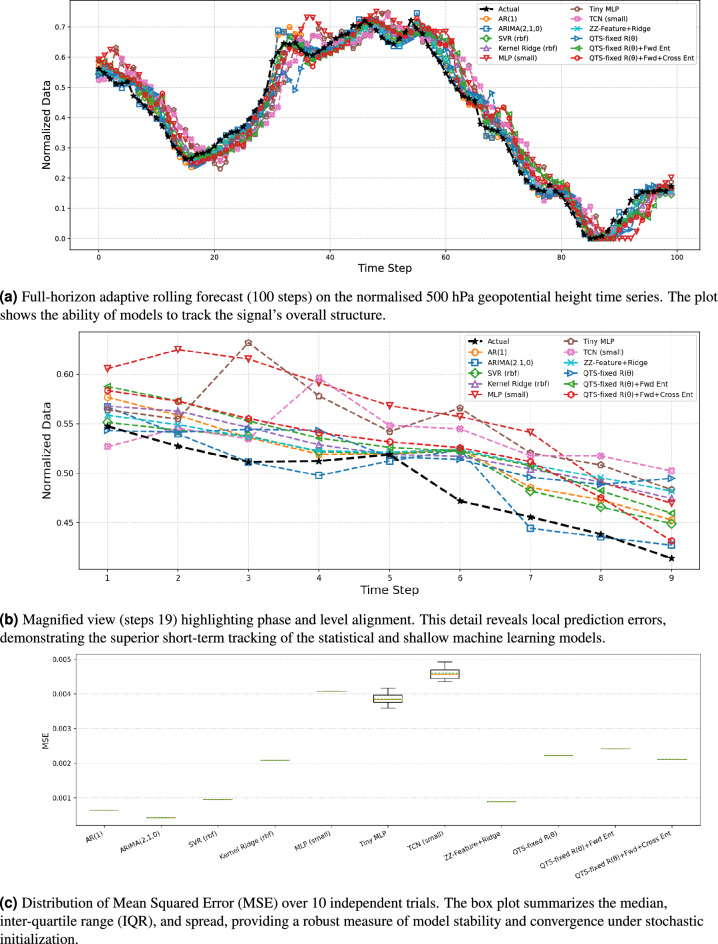
Results for the real atmospheric dataset under a short-window, adaptive rolling forecast. All models are re-fit at each step using a context of ${L=8}$ lags and a minimal training segment of 8 samples. The combined plots illustrate the trade-off between local tracking accuracy and statistical stability.

Figure 3a demonstrates that all predictive models successfully track the essential low-frequency temporal structure of the real atmospheric signal (the normalised $500\text { hPa}$ geopotential height). The critical comparative differences emerge in the local alignment and transient behavior of short-horizon predictions. The magnified view in Fig.  3b (steps 1–9) clarifies these local variations. Lightweight statistical baselines $\textrm{AR}(1)$ and $\textrm{ARIMA}(2,1,0)$ exhibit close phase-lock to the target signal with minimal lag. Among quantum variants, the $\mathrm {ZZ\text {-}Feature+Ridge}$ map and the fixed-parameter $\textrm{QTS}$ families demonstrate stable short-horizon performance with constrained overshoot, achieving accuracy comparable to established shallow machine learning techniques (SVR, Kernel Ridge, and MLP) in this adaptive regime. Conversely, deeper neural forecasters (tiny MLP, TCN) display larger local deviations and phase drift, primarily attributable to the deliberately restricted training context, which prioritizes rapid adaptation over long-range memory retention. To ensure statistical validity, we executed the adaptive rolling-forecast experiment across 10 independent runs with different random seeds. The resulting MSE distribution over the 100-step forecast horizon, summarized in Fig.  3c, captures stochastic variability in initialization and optimization, providing a convergence-independent measure of robustness and stability.

Representative MSE values from a single run include: $\textrm{ARIMA}(2,1,0)\approx 4.26\times 10^{-4}$, $\textrm{AR}(1)\approx 6.39\times 10^{-4}$, and $\mathrm {ZZ\text {-}Feature+Ridge}\approx 8.92\times 10^{-4}$. Among kernel and shallow classical machine learning baselines, $\mathrm {SVR\,(rbf)}\approx 9.51\times 10^{-4}$ and $\mathrm {Kernel\ Ridge\,(rbf)}\approx 2.08\times 10^{-3}$. The fixed-parameter $\textrm{QTS}$ families yielded: $\mathrm {QTS\text {-}fixed}\ R(\theta )\approx 2.22\times 10^{-3}$, $\mathrm {QTS\text {-}fixed}\ R(\theta )+\text {Fwd~Ent}\approx 2.41\times 10^{-3}$, and $\mathrm {QTS\text {-}fixed}\ R(\theta )+\text {Fwd+Cross~Ent}\approx 2.11\times 10^{-3}$. Deeper neural forecasters exhibited comparatively higher errors under the restricted training horizon: $\mathrm {MLP\ (small)}\approx 4.08\times 10^{-3}$, $\mathrm {Tiny\ MLP}\approx 4.17\times 10^{-3}$, and $\mathrm {TCN\ (small)}\approx 4.72\times 10^{-3}$. These results reveal a practical performance trade-off. Low-complexity quantum constructions, particularly $\textrm{R}(\theta )$ layers with nearest-neighbor (and with cross entanglement), and the $\textrm{ZZ}$-feature map, achieve competitive forecasting accuracy while maintaining shallow circuit depth and minimal two-qubit gate overhead. This outcome reinforces the potential of structured, locally entangling quantum layers to provide a scalable and noise-resilient approach to quantum time-series modeling, prioritizing circuit interpretability and robustness over depth-driven expressivity.

### Benchmarking on quantum hardware

To validate the practical viability of the quantum time series model on real quantum devices, we deployed the fixed-parameter QTS circuits with forward and cross entanglement on IBM’s superconducting quantum processors. The primary results were obtained on ibm_kingston (156 qubits, Heron R2 architecture, median two-qubit gate error: $2.081 \times 10^{-3}$) using a 100-qubit circuit for atmospheric time series forecasting. A transpiled circuit for 10 qubits is shown in the supplementary material. As summarized in Table 1, the QTS model with forward and cross entanglement ($R(\theta )+\text {Fwd+Cross Ent}$) achieves MSE $4.32 \times 10^{-4}$ over a 15-step forecast horizon, significantly outperforming deep learning models (TCN: $4.51 \times 10^{-2}$, Transformer: $9.58 \times 10^{-2}$) and performing comparably to classical autoregressive models. Notably, the quantum model achieves this performance using only 100 training points, whereas the classical AR and ARIMA baselines were trained on 1024 samples, demonstrating superior data efficiency. Circuit construction details, including transpilation to native basis gates and execution traces, are provided in the Supplementary Information.

**Table 1:** Mean squared error (MSE) for forecasting models across simulated noisy backends and IBM quantum hardware. The QTS model demonstrates competitive performance and superior data efficiency on real quantum processors, achieving accuracy comparable to classical autoregressive methods despite using significantly fewer training samples.

Model	MSE (CPU and Noisy Backend, 16 steps)	MSE (CPU, and ibm_kingston, 15 steps)
AR(1)	$4.34 \times 10^{-4}$	$4.62 \times 10^{-4}$
AR(2)	$4.00 \times 10^{-4}$	$4.21 \times 10^{-4}$
AR(3)	$4.10 \times 10^{-4}$	$4.31 \times 10^{-4}$
ARIMA(2,1,0)	$4.22 \times 10^{-4}$	$4.16 \times 10^{-4}$
TCN	$5.43 \times 10^{-2}$	$4.51 \times 10^{-2}$
Transformer	$1.22 \times 10^{-1}$	$9.58 \times 10^{-2}$
QTS $R(\theta )$	$1.22 \times 10^{-1}$	—
QTS $R(\theta )+\text {Fwd Ent}$	$1.65 \times 10^{-3}$	—
QTS $R(\theta )+\text {Fwd+Cross Ent}$	$9.20 \times 10^{-4}$	$4.32 \times 10^{-4}$

**Fig. 4:**
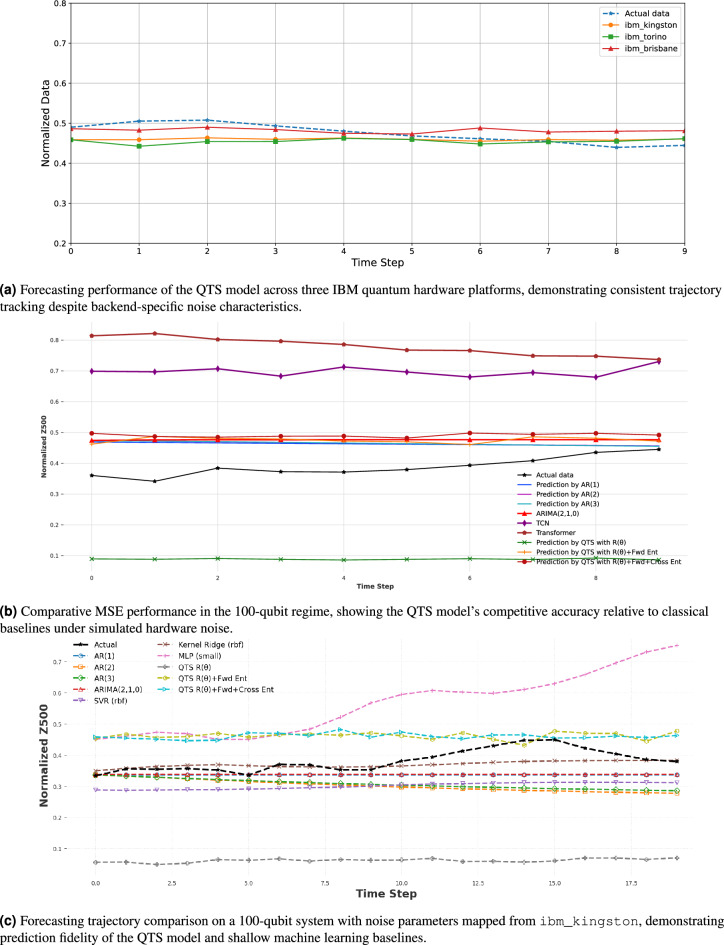
Hardware validation and scalability benchmarks for atmospheric time series forecasting.

To assess robustness across different quantum architectures, we benchmarked the QTS model on three IBM quantum processors: ibm_kingston (156 qubits, Heron R2), ibm_torino (133 qubits, Heron R1), and ibm_brisbane (127 qubits, Eagle R3). As shown in Fig.  4a , all platforms yielded forecasts that closely tracked the actual atmospheric signal despite inherent hardware noise and architectural differences. The $R(\theta )+\text {Fwd+Cross Ent}$ variant demonstrated consistent performance across backends, with minor variations attributable to platform-specific characteristics such as gate fidelity and coherence times. This cross-platform consistency confirms the model’s resilience to device-level noise and its practical applicability across the current generation of IBM quantum processors. Figure 4b presents a comparative analysis of forecasting performance across different model classes in the 100-qubit regime. Traditional statistical models exhibited the lowest MSEs: AR(1) at $6.56 \times 10^{-3}$, AR(2) at $6.97 \times 10^{-3}$, AR(3) at $6.99 \times 10^{-3}$, and ARIMA(2,1,0) at $8.48 \times 10^{-3}$, with these classical models trained on 1024 samples. In contrast, neural network models TCN and Transformer, trained on only 100 data points, showed significantly higher MSEs of $9.63 \times 10^{-2}$ and $1.55 \times 10^{-1}$, respectively, highlighting their limited performance in data-scarce regimes. The QTS model, also trained on 100 data points, demonstrated progressive improvement with increasing entanglement structure: the basic $R(\theta )$ configuration yielded MSE $9.12 \times 10^{-2}$, while incorporating forward entanglement ($R(\theta )+\text {Fwd Ent}$) dramatically improved performance to $8.38 \times 10^{-3}$, approaching classical autoregressive accuracy despite the reduced training set. The fully entangled variant ($R(\theta )+\text {Fwd+Cross Ent}$) achieved MSE $1.12 \times 10^{-2}$, representing a slight increase attributable to accumulated hardware noise and decoherence effects in the longer circuit depth, yet remaining competitive with statistical baselines and substantially superior to deep learning models under data-limited conditions.

To further test scalability, we simulated a 100-qubit system with noise parameters mapped from the ibm_kingston processor, training on 100 points and forecasting 20 steps ahead. Results in Figure 4c demonstrate that the model maintains high trajectory fidelity even at this scale. The kernel ridge regression baseline achieved the lowest MSE ($9.22 \times 10^{-4}$), followed by ARIMA ($3.10 \times 10^{-3}$) and AR(1) ($3.23 \times 10^{-3}$). The fully entangled QTS model ($R(\theta )+\text {Fwd+Cross Ent}$) performed competitively with MSE $7.46 \times 10^{-3}$, maintaining close alignment with the actual signal as visible in the trajectory overlay. This encourages in favor of our intuition that the locally entangled QTS ansatz preserves predictive accuracy and noise resilience at scale, providing a practical approach for quantum time series analysis in regimes.

### Circuit depth and complexity analysis

The quantitative comparison of circuit depth and two-qubit gate (CNOT) counts across various ansatz architectures is summarized in Table 2, spanning up to 20 qubits. Following complete transpilation and decomposition to the native IBM basis set $\texttt {[rz, sx, x, cx]}$, the distinct scaling behavior of each model becomes evident. Amplitude encoding, while offering exponential data capacity ($O(2^n)$ complex amplitudes in *n* qubits), incurs commensurate exponential circuit cost. This is reflected in its gate counts, which could only be decomposed exactly up to 12 qubits before requiring approximation. Highly entangled schemes such as Heisenberg spin-chain encoding and parametric random-unitary ansatz exhibit rapidly increasing circuit depth and CNOT count, consistent with their non-local interaction structure. While such architectures offer enhanced expressive power, this complexity imposes considerable hardware overhead and increases susceptibility to noise, limiting near-term implementability. In contrast, structured low-depth models, particularly the $R(\theta )$-based fixed-parametric QTS families, display markedly slower near-linear growth in complexity with system size *n*. These phase encoding methods embed one real parameter per qubit (*O*(*n*) data dimensionality), with local entanglement introducing pairwise correlations that scale linearly in qubit number.

**Table 2:** Circuit complexity scaling of various quantum ansatz architectures after transpilation and decomposition to the native basis set $\texttt {[rz, sx, x, cx]}$. *Ent.* denotes Entanglement. The amplitude encoding method, which offers exponential data capacity, incurs exponential circuit cost; its exact decomposition was limited to 12 qubits (*approximated beyond $n=12$). The single-qubit $R(\theta )$ layer serves as the minimum complexity baseline, exhibiting constant depth ($\approx 5$) regardless of qubit count and requiring no CNOT gates.

Method / Qubit size (*n*)	2	4	6	8	10	12	14	16	18	20
Circuit depth										
Amplitude encoding (exact/approx*)	12	72	348	1488	6084	24504	16384	65536	262144	1048576
Heisenberg spin-chain encoding	21	63	105	147	189	231	273	315	357	399
Parametric random unitary ansatz	21	28	39	37	48	59	56	64	73	75
Haar-like random circuit	8	10	11	11	11	12	14	13	12	14
$R(\theta )$ + Up to 4-neighbor Ent.	6	10	13	16	18	20	22	24	26	28
$R(\theta )$ + Up to 3-neighbor Ent.	6	10	12	15	17	19	21	23	25	27
$R(\theta )$ + cross (next-nearest) Ent.	6	9	11	13	15	17	19	21	23	25
$R(\theta )$ + nearest-neighbor Ent.	6	8	10	12	14	16	18	20	22	24
ZZ-feature map encoding	8	17	23	29	35	41	47	53	59	65
Single-qubit $R(\theta )$ layer	5	5	5	5	5	5	5	5	5	5
CNOT count										
Amplitude encoding (exact/approx*)	1	11	57	247	1013	4083	16383	65535	262143	1048575
Heisenberg spin-chain encoding	6	18	30	42	54	66	78	90	102	114
Parametric random unitary ansatz	3	10	16	22	28	34	40	46	52	58
Haar-like random circuit	1	4	6	8	10	12	14	16	18	20
$R(\theta )$ + Up to 4-neighbor Ent.	1	6	14	22	30	38	46	54	62	70
$R(\theta )$ + Up to 3-neighbor Ent.	1	6	12	18	24	30	36	42	48	54
$R(\theta )$ + cross (next-nearest) Ent.	1	5	9	13	17	21	25	29	33	37
$R(\theta )$ + nearest-neighbor Ent.	1	3	5	7	9	11	13	15	17	19
ZZ-feature map encoding	2	10	18	26	34	42	50	58	66	74
Single-qubit $R(\theta )$ layer	-	-	-	-	-	-	-	-	-	-

**Fig. 5:**
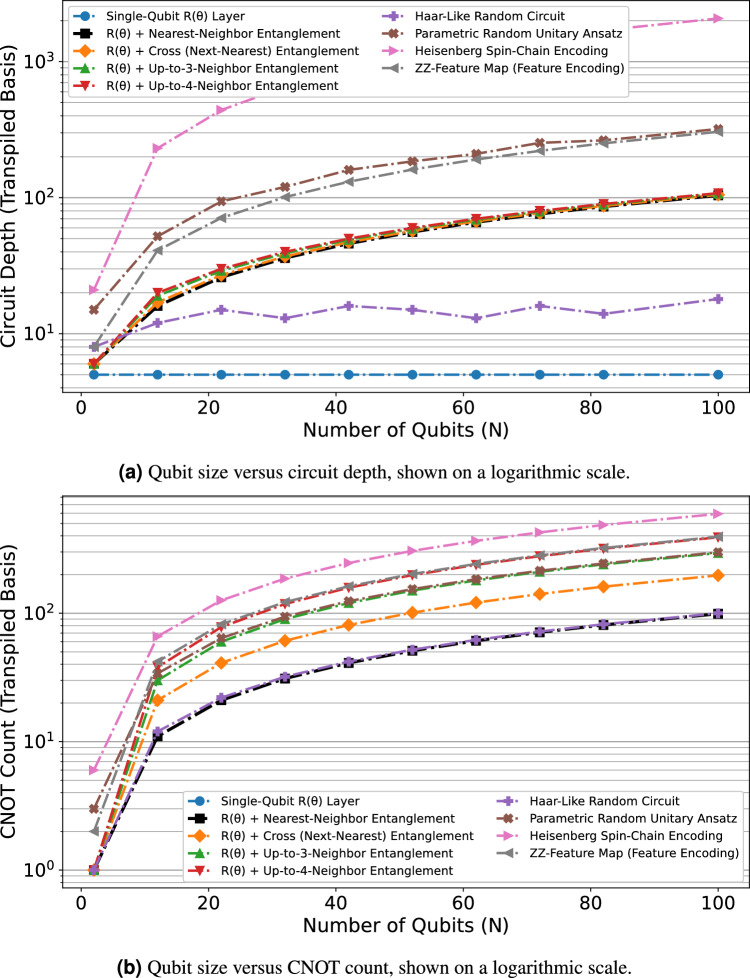
Complexity scaling of various quantum ansatz circuits after transpilation to the basis $\texttt {[rz, sx, x, cx]}$. The plots illustrate performance-limiting resource consumption: (**a**) Circuit depth, and (**b**) CNOT count, both displayed on logarithmic scales to highlight exponential versus linear scaling regimes.

Figure 5 visually reinforces these trends, contrasting the exponential growth characteristic of deep fully entangling circuits with the near-linear scaling observed for low-depth constructions, simulated the proposed method up to 100 qubits. The logarithmic scale representation in Fig.  5a, and b, reveals distinct scaling regimes. As amplitude encoding exhibits the steepest ascent with qubit size reflecting its exponential overhead, we could not show them in the figure. Heisenberg spin-chain and ZZ-feature map encodings follow intermediate trajectories, with depths and gate counts in the range of $10^2$ at large qubit numbers, consistent with their polynomial but superlinear scaling. The cross entanglement variant (including both nearest and next-nearest couplings) exhibits depth near 100 and CNOT count approximately 200 at $n=100$, representing a controlled doubling relative to nearest-neighbor-only connectivity. Even the 3-neighbor and 4-neighbor variants remain within modest resource requirements, with depths below 110 and CNOT counts below 400 at $n=100$. The Haar-like random circuit displays irregular but bounded scaling, remaining below depth 20 across the range due to its sparse random connectivity. These results demonstrate that carefully structured locally entangled parameterized layers offer favorable balance between expressibility and hardware efficiency, with the nearest-neighbor variant providing the scalable architecture for near-term quantum hardware implementation.

### Note on limitations and future work

The QTS framework developed in this study focuses on shallow, hardware-efficient circuit architectures tailored for near-term quantum processors, prioritizing experimental feasibility and interpretability over exhaustive model generality. The present work does not attempt full state-preparation for amplitude-compressed encoding owing to their exponential circuit cost, nor an extensive hyperparameter optimization for large deep-learning baselines, which are left for future exploration. Moreover, while the reported 100-qubit simulations employ realistic noise models mapped from current devices, comprehensive error-mitigation and multivariate, geographically diverse forecasting experiments are reserved for subsequent studies. In future extensions, the QTS architecture can be expanded toward analyzing short- and long-term memory capacities, analogous to quantum reservoir computing (QRC) frameworks, by incorporating deeper entangled circuits to capture richer temporal dependencies in complex dynamical data.

## Discussion and outlook

The entanglement-based Quantum Time Series (QTS) framework presented in this study offers a compact and interpretable approach to forecasting temporal data using near-term quantum hardware. By encoding scalar inputs through single-qubit rotations and incorporating fixed-depth, structured entanglement, the proposed model efficiently captures temporal dependencies while maintaining a shallow circuit footprint. Benchmark results on both synthetic and real geophysical datasets indicate that the framework can potentially achieve performance comparable to well-established classical and shallow machine learning models. Importantly, this is accomplished with modest training data requirements and a limited number of fixed-parameters. The parametric variant can also be exploited in the hybrid quantum-classical to benchmark with a deeper circuit. The demonstrated scalability where circuit depth and two-qubit gate count increase linearly with qubit number supports the view that structured, locally entangled ansatz circuits provide a practical balance between expressibility and hardware feasibility. The experimental execution on recent IBM Quantum processors (e.g., ibm_kingston) further validates the stability of these architectures under realistic noise conditions, highlighting their potential as testbeds for near-term quantum forecasting tasks. While the observed improvements are modest in absolute terms, these suggest that quantum-enhanced temporal modeling is achievable even within current hardware constraints.

Looking ahead, the QTS architecture establishes a foundation for several promising extensions. Incorporating adaptive or trainable entanglement patterns and richer quantum feature maps may enable the model to handle more complex nonlinear or multi-dimensional time series. Embedding physical priors such as conservation constraints or symmetry principles directly into the circuit design could also enhance interpretability for domain-specific applications in physics, chemistry, and finance. Furthermore, integrating QTS with quantum reservoir computing or hybrid quantum–classical recurrent frameworks may expand its applicability to online or streaming data scenarios.

## Supplementary Information

Supplementary Information.

## Data Availability

The datasets supporting the current study are generated synthetically, and an example on open-source data is provided. This is discussed and cited in the main manuscript.

## Code availability

The code generated during the current study is available from the corresponding author upon reasonable request.

## References

[1] Bauer, P., Thorpe, A. & Brunet, G. The quiet revolution of numerical weather prediction. *Nature* **525**, 47–55 (2015). 26333465 10.1038/nature14956

[2] Schultz, M. G. et al. Can deep learning beat numerical weather prediction?. *Phil. Trans. R. Soc. A* **379**, 20200097 (2021). 33583266 10.1098/rsta.2020.0097 PMC7898133

[3] Ollitrault, P. J., Miessen, A. & Tavernelli, I. Molecular quantum dynamics: a quantum computing perspective. *Acc. Chem. Res.* **54**, 4229–4238 (2021). 34787398 10.1021/acs.accounts.1c00514

[4] Box, G. E., Jenkins, G. M., Reinsel, G. C. & Ljung, G. M. *Time Series Anal. Forecast. Control* (John Wiley & Sons, 2015).

[5] Graves, A. Long short-term memory. *Supervised Seq. Labell. Recurr. Neural Netw.* 37–45 (2012).

[6] Cho, K. et al. Learning phrase representations using rnn encoder-decoder for statistical machine translation. arXiv preprint arXiv:1406.1078 (2014).

[7] Lea, C., Flynn, M. D., Vidal, R., Reiter, A. & Hager, G. D. Temporal convolutional networks for action segmentation and detection. In *Proceedings of the IEEE Conference on Computer Vision and Pattern Recognition*, 156–165 (2017).

[8] Bai, S., Kolter, J. Z. & Koltun, V. An empirical evaluation of generic convolutional and recurrent networks for sequence modeling. arxiv. arXiv preprint arXiv:1803.01271 **10** (2018).

[9] Vaswani, A. et al. Attention is all you need. *Adv. Neural Inform. Process. Syst.* **30** (2017).

[10] Lim, B. & Zohren, S. Time-series forecasting with deep learning: a survey. *Phil. Trans. R. Soc. A* **379**, 20200209 (2021). 33583273 10.1098/rsta.2020.0209

[11] Benedetti, M., Lloyd, E., Sack, S. & Fiorentini, M. Parameterized quantum circuits as machine learning models. *Quant. Sci. Technol.* **4**, 043001 (2019).

[12] Sim, S., Johnson, P. D. & Aspuru-Guzik, A. Expressibility and entangling capability of parameterized quantum circuits for hybrid quantum-classical algorithms. *Adv. Quant. Technol.* **2**, 1900070 (2019).

[13] McClean, J. R., Boixo, S., Smelyanskiy, V. N., Babbush, R. & Neven, H. Barren plateaus in quantum neural network training landscapes. *Nat. Commun.* **9**, 4812 (2018). 30446662 10.1038/s41467-018-07090-4 PMC6240101

[14] Cerezo, M. et al. Variational quantum algorithms. *Nat. Rev. Phys.* **3**, 625–644 (2021).

[15] Schuld, M. & Petruccione, F. Supervised learning with quantum computers. *Quant. Sci. Technol.* **17** (2018).

[16] Choi, S., Chowdhury, T. A. & Yu, K. Quantum Utility-scale error mitigation for quantum quench dynamics in Heisenberg spin chains. arXiv preprint arXiv:2506.20125 (2025).

[17] Mujal, P., Martínez-Peña, R., Giorgi, G. L., Soriano, M. C. & Zambrini, R. Time-series quantum reservoir computing with weak and projective measurements. *npj Quant. Inform.* **9**, 16 (2023).

[18] Monomi, T., Setoyama, W. & Hasegawa, Y. Feedback-enhanced quantum reservoir computing with weak measurements. arXiv preprint arXiv:2503.17939 (2025).

[19] Yu, H. & Zhao, X. Event-based deep reinforcement learning for quantum control. *IEEE Trans. Emerg. Topics Comput. Intell.* **8**, 548–562 (2023).

