# Supplementary material for: Shallow entangled circuits for quantum time series prediction on IBM devices
Source: Sci Rep. 2025 Dec 12;15:43727. doi: 10.1038/s41598-025-28512-6 (PMC12700859; doi:10.1038/s41598-025-28512-6)
Supplement: Supplementary file 1 — Supplementary Information. [file 41598_2025_28512_MOESM1_ESM.pdf]

Supplementary Material
Shallow Entangled Circuits for Quantum Time Series Prediction on IBM Devices

Mostafizur Rahaman Laskar, Richa Goel. *IBM Research Lab, India.*

I. Supplementary results

A. *Open-source Dataset and Circuit Transpilation:*

To evaluate the practical applicability of the proposed Quantum Time Series (QTS) framework, we utilize a benchmark geophysical dataset sourced from the WeatherBench archive [1]. This dataset contains geopotential height fields at the 500 hPa pressure level (Z_{500}) for many years. We extract a univariate sequence by calculating the zonal-mean time series along the equator (latitude $= 0°$), isolating the learning of intrinsic temporal patterns. The dataset is publicly available at https://dataserv.ub.tum.de/s/m1524895.

B. *Implementation in QISKIT*

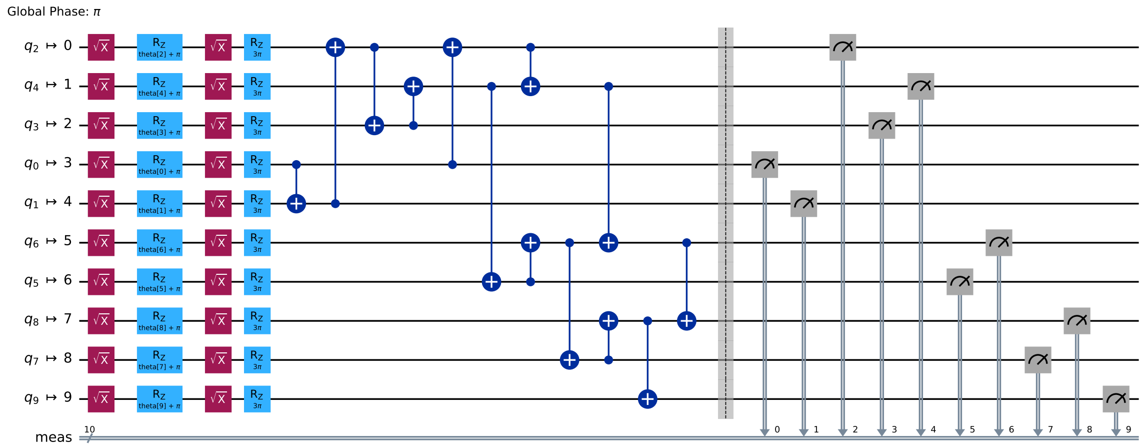

Supplementary Figure 1. Transpiled version of the QTS circuit adapted for IBM hardware. The transpilation maps the circuit to native gates such as $\sqrt{X}$, R_z, and basis-aligned CX operations to match the device connectivity and gate set constraints. Total gate counts after transpilation are 13 CNOTs and 40 single-qubit gates for a 10 qubit circuit, as shown here.

The QTS framework has been implemented in IBM QISKIT platform using python script, with a batch-wise forecasting pipeline. **Supplementary Figure 1.** illustrates fully transpiled circuit used for execution of the QTS algorithm using 10 qubits. The transpilation maps the logically compact QTS design onto the hardware's native gate set ($\sqrt{X}$, R_z, CX) while maintaining the qubit connectivity.

As an example, the **Supplementary Figure 2** is generated with each prediction step using a sliding window over the normalised time series, with $L = 8$ past observations forming the input and one-step-ahead value as the target. The rolling window is advanced sequentially, retraining the quantum model at every iteration using the most recent data segment ($W = 8$ samples). All input values are normalised to the $[0, 1]$ interval before being encoded into qubit rotations through $R_y(2\pi x_i)$ operations. Forecasts are clipped within the same range to preserve scale consistency across steps.

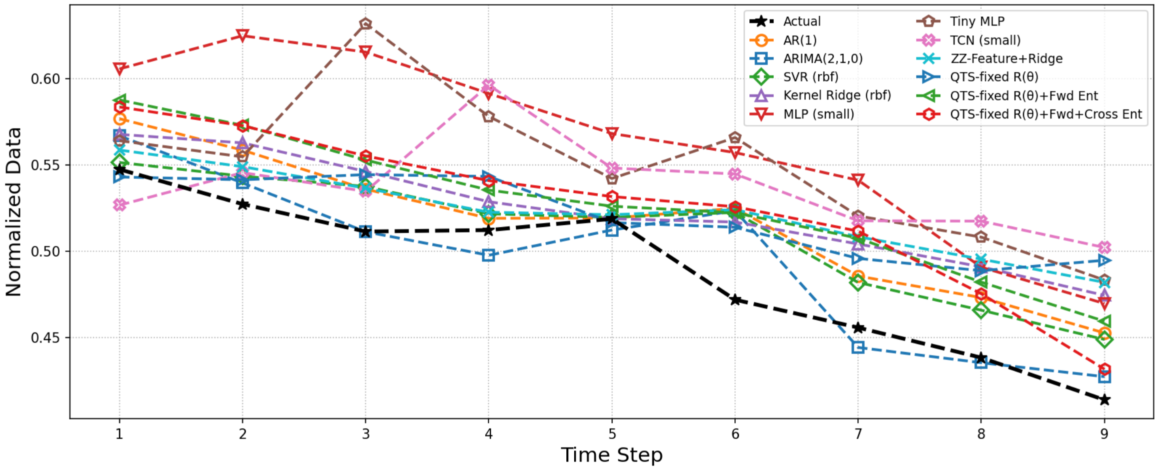

(a) Magnified view (steps 1–9) highlighting phase and level alignment. This detail reveals local prediction errors, demonstrating the superior short-term tracking of the statistical and shallow machine learning models.

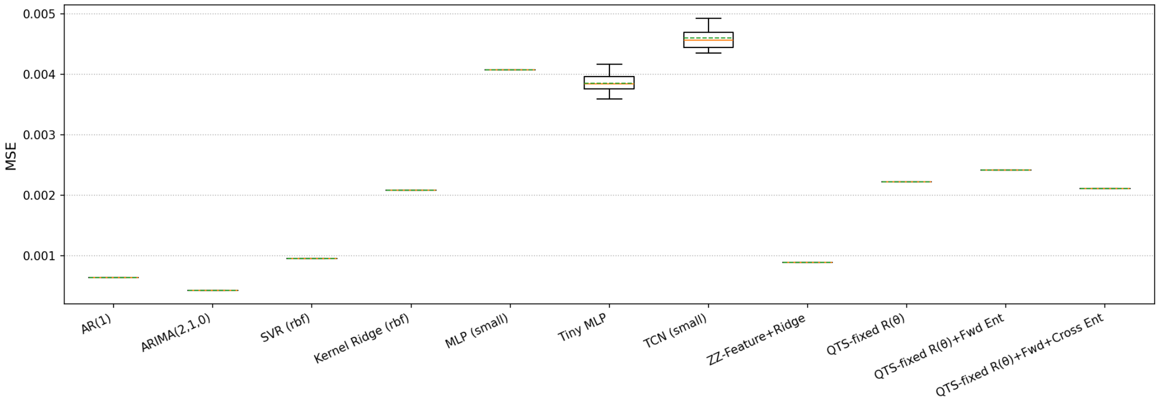

(b) Distribution of Mean Squared Error (MSE) over 10 independent trials. The box plot summarizes the median, interquartile range (IQR), and spread, providing a robust measure of model stability and convergence under stochastic initialization.

Supplementary Figure 2. Results for the Real Atmospheric Dataset under a short-window, adaptive rolling forecast. All models are trained and updated at each step using a context of $L = 8$ time lags and a minimal training segment of 8 samples. The combined plots illustrate the trade-off between local tracking accuracy and statistical stability.

Each QTS variant corresponds to a specific circuit builder implemented within the rolling-forecast loop. The $\mathrm{R}(\theta)$ configuration, referred to as *QTS-fixed* $R(\theta)$, encodes each lagged input component directly as a single-qubit $R_y(\theta_i x_i)$ rotation, where x_i is the normalized time-series value and θ_i a learnable scaling factor. This configuration forms a separable, non-entangled baseline that captures local amplitude variations but no cross-qubit correlations. The $\mathrm{R}(\theta)\mathrm{+Fwd\ Ent}$ model extends this design by appending a unidirectional chain of $\mathrm{CNOT}(i, i{+}1)$ gates across adjacent qubits, creating nearest-neighbor entanglement. The $\mathrm{R}(\theta)\mathrm{+Fwd+Cross\ Ent}$ variant adds an additional layer of $\mathrm{CNOT}(i, i{+}2)$ operations to encode next-nearest-neighbor dependencies, thereby enabling the circuit to capture second-order temporal interactions in the lag window. These entanglement structures were chosen to balance expressivity and hardware efficiency, keeping the depth linear in L while ensuring that no qubit experiences more than two two-qubit interactions per layer.

The parametric circuit (*QTS PARAM*) builds on the same input encoding but augments each qubit with a full rotational block $R_X(\theta_x x_i)\, R_Y(\theta_y x_i)\, R_Z(\theta_z x_i)$ followed by trainable $R_{ZZ}(\phi)$ entanglers connecting neighboring pairs. This circuit family employs four global parameters $(\theta_x, \theta_y, \theta_z, \phi)$ optimized through a classical optimizer, Broyden–Fletcher–Goldfarb–Shanno (BFGS), minimizing the rolling mean-squared loss over the forecast window. For each constructed circuit, the final quantum state $|\psi\rangle$ is evaluated using the `Statevector` simulator in Qiskit, from which the probability amplitudes $P_q(k) = |\langle k|\psi\rangle|^2$ are extracted. A scalar forecast signal is

then computed as the weighted expectation $\sum_k (k/(2^L-1))P_q(k)$, effectively projecting the probability mass function onto a continuous target interval $[0,1]$.

In the ZZ-Feature+Ridge variant, each qubit applies $R_y(2\pi x_i)$ rotations followed by fixed entangling layers consisting of $R_{ZZ}(\pi/4)$ on adjacent qubits $(i, i+1)$ and $R_{ZZ}(\pi/8)$ on next-adjacent pairs $(i, i+2)$. The resulting circuit defines a nonlinear feature map from the input vector $\mathbf{x}$ to a measurement space spanned by the expectation values of single-qubit and two-qubit Pauli-Z operators: $\langle Z_i \rangle$, $\langle Z_i Z_{i+1} \rangle$, and $\langle Z_i Z_{i+2} \rangle$. These features are concatenated and used to fit a ridge regression model $(\mathbf{w}, b)$ in closed form at each forecast step, yielding predictions $\hat{y} = \mathbf{w}^\top \boldsymbol{\phi} + b$.

All models were executed under identical simulation conditions with $L = W = 8$, forecast horizon $S = 100$, and additive Gaussian noise injected at each step for stochastic robustness. Random seeds were systematically offset between trials to generate distinct realizations while preserving reproducibility. The regularization constants were set to $\lambda_\theta = 10^{-2}$ for parametric optimization and $\lambda_{\mathrm{R}} = 10^{-2}$ for ridge fitting, with a cap of 70 BFGS iterations per window. Across all settings, the resulting circuit depths remained between 12 and 18 layers, and the total two-qubit gate count scaled linearly with L. For the large circuits such as 100 qubits, we run the shallow quantum entanglement-based circuits on IBM Hardware (e.g., `ibm_kingston`, `ibm_torino`, `ibm_brisbane`).

Supplementary Figure 2(a) and **Supplementary Figure 2(b)** illustrate the outputs of this implementation. In **Supplementary Figure 2(a)**, the 'QTS-fixed $R(\theta)$', 'QTS-fixed $R(\theta)$+Fwd Ent', and 'QTS-fixed $R(\theta)$+Fwd +Cross Ent' models exhibit stable short-term phase tracking, closely following the $\mathrm{ARIMA}(2,1,0)$ and $\mathrm{AR}(1)$ baselines with minimal lag and amplitude drift. **Supplementary Figure 2(b)** shows the mean-squared error (MSE) distributions over ten independent runs, where the QTS circuits achieve values in the range of $(2.1\text{–}2.4)\times 10^{-3}$, comparable to kernel ridge regression and significantly below the neural baselines (TCN, MLP) with $(4.0\text{–}4.7)\times 10^{-3}$.

C. Sources of Error and Mitigation Strategies

The performance of Quantum Time Series (QTS) models is affected by both classical and quantum noise. Classical noise arises from stochastic fluctuations or measurement uncertainties in the input data, whereas quantum noise originates from hardware imperfections such as gate infidelities, decoherence, crosstalk, and readout errors (however, crosstalk and readout errors are negligible in latest IBM processors such as Heron r3). These effects become increasingly significant with circuit depth and two-qubit gate count, motivating the design of shallow, hardware-efficient architectures as used in our implementation. To alleviate such effects, we employed several pragmatic strategies: (i) constructing low-depth circuits with minimal CNOT layers (ii) performing hardware-aware transpilation through Qiskit's optimization levels to match native gate sets and coupling maps, and (iii) averaging over a higher number of measurement shots to reduce statistical variance. These methods together support the proposed QTS circuits maintain numerical stability and application to near-term quantum devices.

It would be valuable to explore advanced error mitigation and suppression methods on IBM quantum Runtime environment, as a future scope of the work, especially for the parametric variant of the QTS for complex time series forecasting. Techniques such as zero-noise extrapolation (ZNE), and probabilistic error cancellation (PEC) could be systematically explored to enhance the reliability of QTS predictions under realistic noise conditions. Additionally, dynamical decoupling and pulse-level error suppression, supported by IBM hardware, may further stabilize temporal circuit execution, for longer coherent evolutions in parametric QTS versions.

D. Complexity Analysis (Asymptotic)

The QTS framework achieves a marked reduction in computational scaling compared to conventional forecasting models, summarized in **Supplementary Table I**. This efficiency stems from a concise quantum representation where circuit resources scale logarithmically with the original time series length N using phase encoding (QTS with shallow depth circuits), or $\mathcal{O}(\log N)$ (using QTS with amplitude encoding ansatz). The number of parameters in the shallow QTS variants as shown scales linearly with the number of qubits n, i.e., $\mathcal{O}(n)$, while it varies for the parametric variants depending on the depth of the circuit. As the overall gate

complexity is shallow for the presented QTS method (**Supplementary Table I**), scaling as $\mathcal{O}(n)$, it makes the architecture hardware-efficient, and can be scaled to utility regime (100+ qubits). Further, the runtime per forecast step is $\mathcal{O}(M_1 \cdot n)$, where M_1 is the number of measurement shots. The asymptotic behaviour shows that the structured entanglement topology (forward and cross-qubit connections) has a good expressivity within optimal circuit depth.

Model	Training Complexity	Parameter Complexity	Model / Gate Complexity
AR(p)	$\mathcal{O}(Np^2)$	$\mathcal{O}(p)$	$\mathcal{O}(Np^2)$
ARIMA(p, d, q)	$\mathcal{O}(N(p+q)^2)$	$\mathcal{O}(p+q)$	$\mathcal{O}(N(p+q)^2)$
MLP (small)	$\mathcal{O}(ELNd^2)$	$\mathcal{O}(Ld^2)$	$\mathcal{O}(LNd^2)$
TCN [2]	$\mathcal{O}(ELkNd^2)$	$\mathcal{O}(Ld^2)$	$\mathcal{O}(LkNd^2)$
Transformer [3]	$\mathcal{O}(ELN^2d)$	$\mathcal{O}(Ld^2)$	$\mathcal{O}(LN^2d)$
QTS ($R(\theta)$)	$\mathcal{O}(\log N)$	$\mathcal{O}(\log N)$	$\mathcal{O}(M_1 \log N)$
QTS ($R(\theta)$ + Fwd Ent.)	$\mathcal{O}(\log N)$	$\mathcal{O}(\log N)$	$\mathcal{O}(M_1 \log N)$
QTS ($R(\theta)$ + Fwd + Cross)	$\mathcal{O}(\log N)$	$\mathcal{O}(\log N)$	$\mathcal{O}(M_1 \log N)$

Supplementary Table I. Comparative scaling of classical and quantum time series forecasting models. Here N denotes sequence length; p, q, and d are ARIMA orders; L, E, and k represent layer count, training epochs, and kernel size; and M_1 is the number of quantum circuit shots. QTS models exhibit logarithmic dependence on N and linear scaling with qubit count, offering compact, hardware-efficient forecasting performance.

E. Scalability of Various Quantum Ansatz

Supplementary Table II. summarizes the complexity and structure of various quantum ansatz relevant for data encoding and temporal modelling, contrasting the deep complexity of existing schemes with the shallow, structured approach of the QTS framework. The QTS model's superior scalability ($\mathcal{O}(n)$ depth) contrasts favorably with deep, globally entangling schemes like Amplitude Encoding ($\mathcal{O}(2^n)$ depth) and Haar-Random circuits ($\mathcal{O}(n^2)$ depth), confirming its suitability for current and near-term hardware up to systems approaching *100* qubits.

Method	Depth	Structure	Scalability	Remarks
Haar-Random	$\mathcal{O}(n)$ to $\mathcal{O}(n^2)$	Dense, Global	Low	Maximally expressive but deep; unsuitable for large n (Ref. [4])
Heisenberg PQC	$\sim \mathcal{O}(n^2)$	Local, Structured	Moderate	Spin-chain analog; trainable and interpretable [5]
Amplitude Encoding	$\mathcal{O}(2^n)$	Global	Low	Efficient qubit count; exponential depth for generic states [6]
Random Circuit	$\sim \mathcal{O}(n^2)$	Unstructured	Low	Variable performance; poor alignment with temporal tasks [7], [8]
Proposed Model	$\mathcal{O}(n)$	Sparse, Structured	High ($n \sim$ *100*)	Causal, interpretable; shallow circuits ideal for noisy Hardware

Supplementary Table II. Comparison of quantum encoding schemes for time-dependent data. Here, $' \sim '$ denotes approximation for some methods based on experimental data.

F. MSE Performance of Various Quantum Ansatz

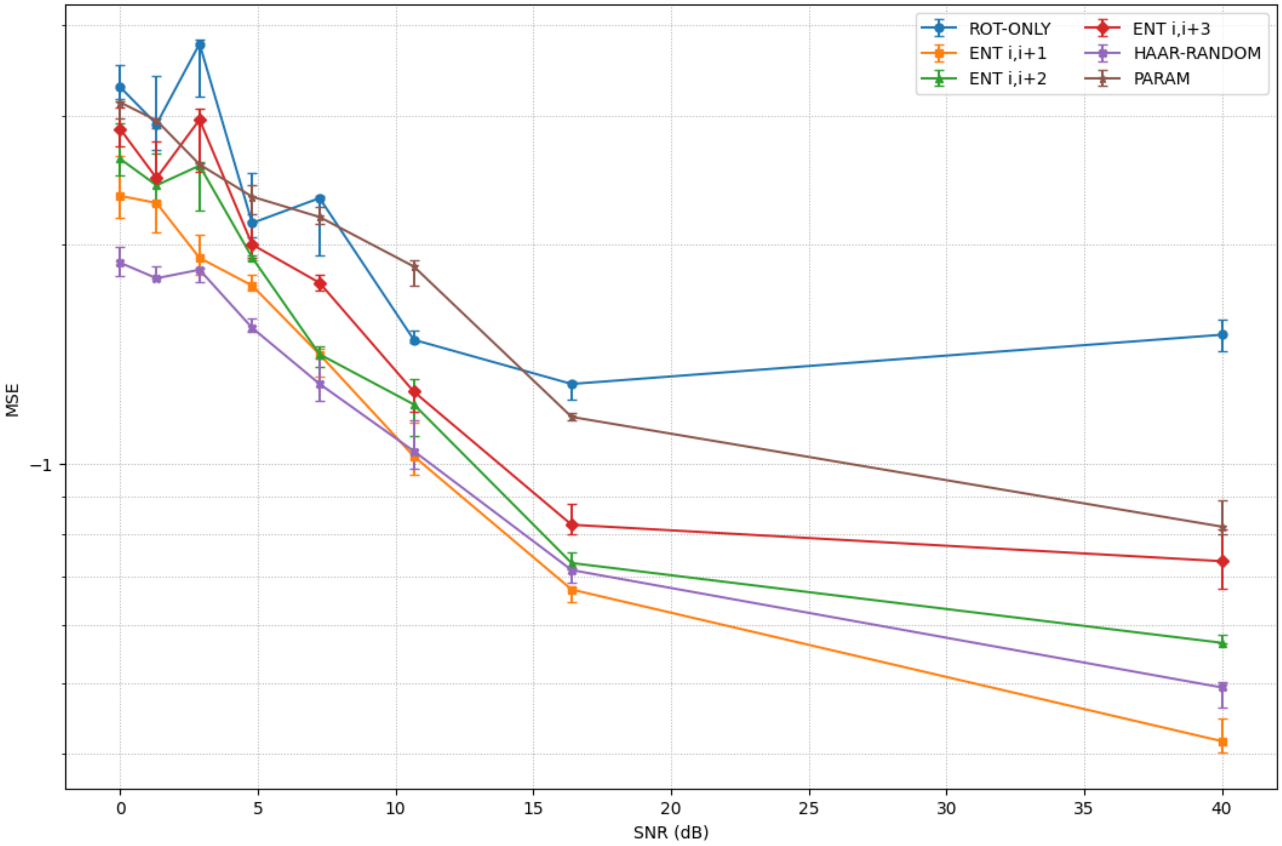

Supplementary Figure 3. Performance comparison (MSE vs. SNR) on the real atmospheric dataset for different entanglement configurations. *ROT-ONLY* denotes the separable baseline ($R_y(\theta)$ only). $\mathrm{ENT\ i, i+1}$ and $\mathrm{ENT\ i, i+2}$ correspond to structured forward and forward+cross entanglement, respectively, while $\mathrm{ENT\ i, i+3}$ represents deeper connectivity (tertiary order of entanglement). $\mathrm{HAAR\text{-}RANDOM}$ indicates Haar-randomized two-qubit couplings, and PARAM refers to the parametric unitary circuit with trainable entangling phases.

Supplementary Figure 3. shows the mean squared error (MSE) variation with signal-to-noise ratio (SNR) across multiple entanglement configurations for the Quantum Time Series (QTS) framework. The results reveal a consistent monotonic decrease in MSE as SNR improves, confirming that all architectures exhibit noise-resilient behavior. The separable $\mathrm{ROT\text{-}ONLY}$ (using only phase gate $R(\theta)$ encoding) circuit retains the highest error floor, highlighting its limited ability to capture inter-temporal correlations. Introducing nearest-neighbor entanglement ($\mathrm{ENT\ i, i+1}$) markedly enhances prediction accuracy, achieving the lowest overall MSE, followed closely by the $\mathrm{ENT\ i, i+2}$ configuration. Both maintain stable variance across the tested SNR range, demonstrating that sparse, causal entanglement suffices for accurate temporal representation. The above result in **Supplementary Figure 3** is generated on 6 qubits QTS circuit, for 100 points using the real dataset. The minimum noise standard deviation was taken as 0.01, with signal power=1, for the SNR.

The inclusion of longer-range coupling ($\mathrm{ENT\ i, i+3}$) or randomized entanglement (Haar-random) yields intermediate performance, with slightly higher error at low SNR and mild degradation in the high-SNR regime, reflecting increased susceptibility to circuit noise and decoherence. The parametric ansatz (PARAM) achieves comparable accuracy at intermediate SNR values but converges more slowly at higher SNR, suggesting potential over-parameterization under limited data. It seems that the structured forward and cross entanglement schemes ($\mathrm{ENT\ i, i+1}$ and $\mathrm{ENT\ i, i+2}$) provide the favorable trade-off between robustness, interpretability, and hardware efficiency for short-term memory based forecasting tasks.

References

[1] S. Rasp, P. D. Dueben, S. Scher, J. A. Weyn, S. Mouatadid, and N. Thuerey, "Weatherbench: a benchmark data set for data-driven weather forecasting," *Journal of Advances in Modeling Earth Systems*, vol. 12, no. 11, p. e2020MS002203, 2020.

[2] S. Bai, J. Z. Kolter, and V. Koltun, "An empirical evaluation of generic convolutional and recurrent networks for sequence modeling. arxiv," *arXiv preprint arXiv:1803.01271*, vol. 10, 2018.

[3] A. Vaswani, N. Shazeer, N. Parmar, J. Uszkoreit, L. Jones, A. N. Gomez, Ł. Kaiser, and I. Polosukhin, "Attention is all you need," *Advances in neural information processing systems*, vol. 30, 2017.

[4] J. Haferkamp, "Random Quantum Circuits are Approximate Unitary t-Designs in Depth $O\left(nt^{5+o(1)}\right)$," *Quantum*, vol. 6, p. 795, 2022.

[5] E. Lötstedt and K. Yamanouchi, "Comparison of encoding schemes for quantum computing of s> 1/2 spin chains," *Physical Review A*, vol. 111, no. 6, p. 062416, 2025.

[6] N. Mitsuda, T. Ichimura, K. Nakaji, Y. Suzuki, T. Tanaka, R. Raymond, H. Tezuka, T. Onodera, and N. Yamamoto, "Approximate complex amplitude encoding algorithm and its application to data classification problems," *Physical Review A*, vol. 109, no. 5, p. 052423, 2024.

[7] Y. Che, C. Gneiting, X. Wang, and F. Nori, "Quantum circuit complexity and unsupervised machine learning of topological order," *arXiv preprint arXiv:2508.04486*, 2025.

[8] D.-L. Deng, X. Li, and S. Das Sarma, "Quantum entanglement in neural network states," *Physical Review X*, vol. 7, no. 2, p. 021021, 2017.
